# 2,4-Diamino-6-methyl-1,3,5-triazin-1-ium hydrogen oxalate

**DOI:** 10.1107/S1600536812016637

**Published:** 2012-04-21

**Authors:** Leila Narimani, Bohari M. Yamin

**Affiliations:** a School of Chemical Sciences and Food Technology, Universiti Kebangsaan Malaysia, 43600 Bangi, Selangor, Malaysia

## Abstract

The title compound, C_4_H_8_N_5_^+^·C_2_HO_4_^−^, was obtained from the reaction of oxalic acid and 2,4-diamino-6-methyl-1,3,5-triazine. The protonated triazine ring is essentially planar with a maximum deviation of 0.035 (1) Å, but the hydrogen oxalate anion is less planar, with a maximum deviation of 0.131 (1) Å for both carbonyl O atoms. In the crystal, the ions are linked by inter­molecular N—H⋯O, N—H⋯N, O—H⋯O and C—H⋯O hydrogen bonds, forming a three-dimensional network. Weak π–π [centroid–centroid distance = 3.763 Å] and C—O⋯π inter­actions [O⋯centroid = 3.5300 (16) Å, C—O⋯centroid = 132.19 (10)°] are also present.

## Related literature

For bond-length data see: Allen *et al.* (1987 ▶) and for a description of the Cambridge Structural Database, see: Allen (2002 ▶). For background to triazine derivatives, see: Sebenik *et al.* (1989 ▶). For related structures, see: Kaczmarek *et al.* (2008 ▶); Xiao (2008 ▶); Fan *et al.* (2009 ▶); Qian & Huang (2010 ▶).
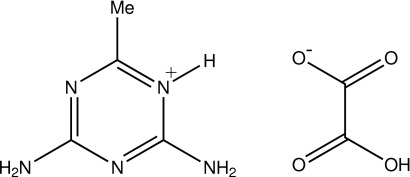


## Experimental

### 

#### Crystal data

C_4_H_8_N_5_^+^·C_2_HO_4_^−^
*M* *_r_* = 215.18
Triclinic, 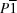

*a* = 5.6208 (12) Å
*b* = 7.9828 (17) Å
*c* = 10.857 (2) Å
α = 76.846 (4)°
β = 75.882 (4)°
γ = 75.954 (4)°
*V* = 450.92 (17) Å^3^
*Z* = 2
Mo *K*α radiation
μ = 0.13 mm^−1^
*T* = 298 K
0.50 × 0.22 × 0.19 mm

#### Data collection

Bruker SMART APEX CCD area-detector diffractometer
Absorption correction: multi-scan (*SADABS*; Sheldrick, 1996 ▶) *T*_min_ = 0.935, *T*_max_ = 0.974
5551 measured reflections
1959 independent reflections
1708 reflections with *I* > 2σ(*I*)
*R*_int_ = 0.023

#### Refinement

*R*[*F* ^2^ > 2σ(*F* ^2^)] = 0.041
*wR*(*F* ^2^) = 0.113
*S* = 1.03
1959 reflections
145 parameters
1 restraint
H atoms treated by a mixture of independent and constrained refinement
Δρ_max_ = 0.25 e Å^−3^
Δρ_min_ = −0.27 e Å^−3^

### 

Data collection: *SMART* (Siemens, 1996 ▶); cell refinement: *SAINT* (Siemens, 1996 ▶); data reduction: *SAINT*; program(s) used to solve structure: *SHELXS97* (Sheldrick, 2008 ▶); program(s) used to refine structure: *SHELXL97* (Sheldrick, 2008 ▶); molecular graphics: *SHELXTL* (Sheldrick, 2008 ▶); software used to prepare material for publication: *SHELXTL*, *PARST* (Nardelli, 1995 ▶) and *PLATON* (Spek, 2009 ▶).

## Supplementary Material

Crystal structure: contains datablock(s) global, I. DOI: 10.1107/S1600536812016637/zq2157sup1.cif

Structure factors: contains datablock(s) I. DOI: 10.1107/S1600536812016637/zq2157Isup2.hkl

Supplementary material file. DOI: 10.1107/S1600536812016637/zq2157Isup3.cml

Additional supplementary materials:  crystallographic information; 3D view; checkCIF report

## Figures and Tables

**Table 1:** Hydrogen-bond geometry (Å, °)

*D*—H⋯*A*	*D*—H	H⋯*A*	*D*⋯*A*	*D*—H⋯*A*
N3—H3⋯O1^i^	0.95 (2)	1.77 (2)	2.7134 (17)	174 (2)
N3—H3⋯O4^i^	0.95 (2)	2.50 (2)	2.9841 (17)	111.7 (16)
O4—H4⋯O2	0.83 (2)	1.66 (2)	2.4921 (16)	175 (2)
N4—H4*D*⋯O3^ii^	0.86	2.17	2.9902 (19)	160
N4—H4*E*⋯N1^iii^	0.86	2.18	3.0399 (19)	174
N5—H5*A*⋯N2^iv^	0.86	2.14	3.0027 (19)	179
N5—H5*B*⋯O2^v^	0.86	2.28	2.8558 (17)	124
N5—H5*B*⋯O3^v^	0.86	2.59	3.2337 (19)	133
C4—H4*C*⋯O1^vi^	0.96	2.49	3.339 (2)	148

Symmetry codes: (i) 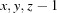; (ii) 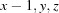; (iii) 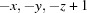; (iv) 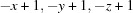; (v) 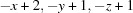; (vi) 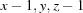.

**Fig. 1.**
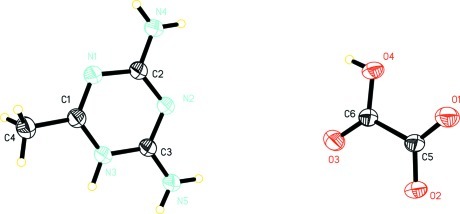
Molecular structure of the title compound with 50% probability displacement ellipsoids.

**Fig. 2.**
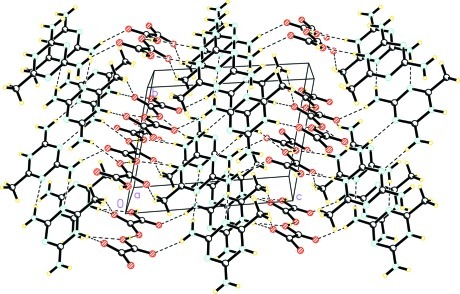
Packing diagram of the title compound viewed down a axis. The dashed lines denote hydrogen bonds.

## supplementary crystallographic information

### Comment

It is known that many triazine derivatives possess biological properties beside its usefullness as intermediates in the pharmaceutical industry (Sebenik *et al.*, 1989). 2,4-diamino-6-1,3,5-triazine has been reported to co-crystallize with methanol (Kaczmarek *et al.*, 2008) and ethanol (Xiao, 2008). On the other hand, the triazine nitrogen atom at position 1 can be easily protonated as in compound (C_4_H_8_N_5_)Cl (Qian & Huang, 2010) and (C_4_H_8_N_5_)NO_3_ (Fan *et al.*, 2009) which were obtained from the normal acid-base reaction. The title compound is a similar salt but having a hydrogen oxalate as counter anion (Fig.1). The non hydrogen triazine ring, C1/N1/C2/N2/C3/N3, is planar with a maximum deviation of 0.035 (1) Å from the least square plane for N3 atom. The hydrogen oxalate anion O1/C1/O2/C2/O3/O4, is less planar with a maximum deviation of 0.131 (1) Å for O1 and O4 atoms. The bond lengths and angles are in normal ranges (Allen *et al.*, 1987; Allen, 2002). In the crystal structure the molecules are linked by N—H···O, N—H···N, O—H···O and C—H···O intermolecular hydrogen bonds (symmetry codes as in Table 2) to form a three-dimensional network (Fig. 2). In addition, there are weak π–π interactions between the triazine ring centroids *Cg*1 (symmetry code: 1-*x*, -*y*, 1-*z*) with a distance of 3.763 Å and a C6—O3···π involving the triazine (C1/N1/C2/N2/C3/N3) centroid (symmetry code: 1-*x*, 1-*y*, 1-*z*) with a O3···*Cg*1 distance of 3.5300 (16) Å and a C6—O3—*Cg*1 bond angle of 132.19 (10)°.

### Experimental

10 ml aqueous solution of ammonium thiocyanate (0.152 g, 2 mmol) was added into a beaker containing oxalate acid (0.126 g, 1 mmol) and 2,4-diamino- 6-methyl-1,3,5-triazine (2 mmol) in 40 ml distilled water. After one week of evaporation at room temperature colourless crystals were obtained. Yield 92%; Melting point: 457.1–458.3 K.

### Refinement

After their location in the difference map, the H-atoms attached to the C and the amino N atoms were fixed geometrically at ideal positions and allowed to ride on the parent atoms with C—H = 0.93 Å and N—H = 0.86 Å, and with *U*_iso_(H) = 1.5*U*_eq_(C) and 1.2*U*_eq_(N). However, the protonated amino and hydroxyl hydrogen atoms were located from the Fourier map and refined isotropically.

### Crystal data

**Table:**

C_4_H_8_N_5_^+^·C_2_HO_4_^−^	*Z* = 2
*M**_r_* = 215.18	*F*(000) = 224
Triclinic, *P*1	*D*_x_ = 1.585 Mg m^−^^3^
Hall symbol: -P 1	Melting point = 492.2–492.5 K
*a* = 5.6208 (12) Å	Mo *K*α radiation, λ = 0.71073 Å
*b* = 7.9828 (17) Å	Cell parameters from 1963 reflections
*c* = 10.857 (2) Å	θ = 1.9–27.0°
α = 76.846 (4)°	µ = 0.13 mm^−^^1^
β = 75.882 (4)°	*T* = 298 K
γ = 75.954 (4)°	Block, colourless
*V* = 450.92 (17) Å^3^	0.50 × 0.22 × 0.19 mm

### Data collection

**Table:**

Bruker SMART APEX CCD area-detector diffractometer	1959 independent reflections
Radiation source: fine-focus sealed tube	1708 reflections with *I* > 2σ(*I*)
Graphite monochromator	*R*_int_ = 0.023
Detector resolution: 83.66 pixels mm^-1^	θ_max_ = 27.0°, θ_min_ = 1.9°
ω scan	*h* = −7→7
Absorption correction: multi-scan (*SADABS*; Sheldrick, 1996)	*k* = −10→10
*T*_min_ = 0.935, *T*_max_ = 0.974	*l* = −13→13
5551 measured reflections	

### Refinement

**Table:**

Refinement on *F*^2^	Primary atom site location: structure-invariant direct methods
Least-squares matrix: full	Secondary atom site location: difference Fourier map
*R*[*F*^2^ > 2σ(*F*^2^)] = 0.041	Hydrogen site location: inferred from neighbouring sites
*wR*(*F*^2^) = 0.113	H atoms treated by a mixture of independent and constrained refinement
*S* = 1.03	*w* = 1/[σ^2^(*F*_o_^2^) + (0.0664*P*)^2^ + 0.0976*P*] where *P* = (*F*_o_^2^ + 2*F*_c_^2^)/3
1959 reflections	(Δ/σ)_max_ = 0.001
145 parameters	Δρ_max_ = 0.25 e Å^−^^3^
1 restraint	Δρ_min_ = −0.27 e Å^−^^3^

### Special details

**Table:**

Geometry. All e.s.d.'s (except the e.s.d. in the dihedral angle between two l.s. planes) are estimated using the full covariance matrix. The cell e.s.d.'s are taken into account individually in the estimation of e.s.d.'s in distances, angles and torsion angles; correlations between e.s.d.'s in cell parameters are only used when they are defined by crystal symmetry. An approximate (isotropic) treatment of cell e.s.d.'s is used for estimating e.s.d.'s involving l.s. planes.
Refinement. Refinement of *F*^2^ against ALL reflections. The weighted *R*-factor *wR* and goodness of fit *S* are based on *F*^2^, conventional *R*-factors *R* are based on *F*, with *F* set to zero for negative *F*^2^. The threshold expression of *F*^2^ > σ(*F*^2^) is used only for calculating *R*-factors(gt) *etc*. and is not relevant to the choice of reflections for refinement. *R*-factors based on *F*^2^ are statistically about twice as large as those based on *F*, and *R*- factors based on ALL data will be even larger.

### Fractional atomic coordinates and isotropic or equivalent isotropic displacement parameters (Å^2^)

**Table:**

	*x*	*y*	*z*	*U*_iso_*/*U*_eq_	
O1	1.01323 (19)	0.22067 (16)	1.09284 (10)	0.0435 (3)	
O2	1.20977 (17)	0.36561 (14)	0.91142 (9)	0.0383 (3)	
O3	0.8189 (2)	0.38620 (18)	0.80042 (10)	0.0490 (3)	
O4	0.60164 (18)	0.30135 (15)	0.99698 (10)	0.0401 (3)	
H4	0.476 (3)	0.322 (3)	0.964 (2)	0.076 (7)*	
N1	0.2651 (2)	0.08398 (15)	0.40173 (11)	0.0342 (3)	
N2	0.3693 (2)	0.31924 (15)	0.46944 (11)	0.0326 (3)	
N3	0.5937 (2)	0.21615 (15)	0.28003 (11)	0.0324 (3)	
H3	0.733 (4)	0.221 (2)	0.2103 (19)	0.049 (5)*	
N4	0.0411 (2)	0.18887 (17)	0.58245 (12)	0.0421 (3)	
H4D	0.0102	0.2572	0.6377	0.051*	
H4E	−0.0506	0.1130	0.5922	0.051*	
N5	0.7025 (2)	0.43757 (17)	0.34456 (12)	0.0393 (3)	
H5A	0.6827	0.5064	0.3983	0.047*	
H5B	0.8206	0.4415	0.2771	0.047*	
C1	0.4493 (3)	0.09606 (17)	0.30219 (13)	0.0307 (3)	
C2	0.2280 (3)	0.20042 (18)	0.48339 (13)	0.0316 (3)	
C3	0.5530 (3)	0.32675 (18)	0.36537 (12)	0.0302 (3)	
C4	0.5070 (3)	−0.0268 (2)	0.21033 (15)	0.0400 (4)	
H4A	0.5078	−0.1442	0.2574	0.060*	
H4B	0.6685	−0.0204	0.1560	0.060*	
H4C	0.3822	0.0048	0.1580	0.060*	
C5	1.0268 (2)	0.30348 (18)	0.98158 (13)	0.0296 (3)	
C6	0.8009 (2)	0.33563 (18)	0.91548 (13)	0.0301 (3)	

### Atomic displacement parameters (Å^2^)

**Table:**

	*U*^11^	*U*^22^	*U*^33^	*U*^12^	*U*^13^	*U*^23^
O1	0.0288 (5)	0.0646 (7)	0.0332 (6)	−0.0124 (5)	−0.0051 (4)	0.0009 (5)
O2	0.0237 (5)	0.0565 (7)	0.0344 (6)	−0.0147 (4)	−0.0014 (4)	−0.0052 (5)
O3	0.0351 (6)	0.0828 (9)	0.0297 (6)	−0.0157 (6)	−0.0046 (4)	−0.0093 (5)
O4	0.0234 (5)	0.0622 (7)	0.0351 (6)	−0.0161 (5)	−0.0038 (4)	−0.0035 (5)
N1	0.0374 (7)	0.0359 (6)	0.0315 (6)	−0.0161 (5)	0.0015 (5)	−0.0103 (5)
N2	0.0381 (6)	0.0360 (6)	0.0262 (6)	−0.0158 (5)	0.0009 (5)	−0.0091 (5)
N3	0.0344 (6)	0.0375 (6)	0.0265 (6)	−0.0140 (5)	0.0022 (5)	−0.0097 (5)
N4	0.0467 (7)	0.0500 (8)	0.0340 (7)	−0.0264 (6)	0.0091 (5)	−0.0165 (6)
N5	0.0447 (7)	0.0487 (7)	0.0297 (6)	−0.0263 (6)	0.0048 (5)	−0.0124 (5)
C1	0.0338 (7)	0.0308 (6)	0.0282 (7)	−0.0090 (5)	−0.0039 (5)	−0.0061 (5)
C2	0.0353 (7)	0.0343 (7)	0.0263 (7)	−0.0126 (6)	−0.0019 (5)	−0.0064 (5)
C3	0.0338 (7)	0.0337 (7)	0.0241 (6)	−0.0117 (5)	−0.0031 (5)	−0.0045 (5)
C4	0.0471 (9)	0.0389 (8)	0.0360 (8)	−0.0132 (6)	0.0003 (6)	−0.0147 (6)
C5	0.0222 (6)	0.0375 (7)	0.0291 (7)	−0.0064 (5)	−0.0002 (5)	−0.0110 (5)
C6	0.0247 (6)	0.0381 (7)	0.0289 (7)	−0.0084 (5)	−0.0013 (5)	−0.0103 (5)

### Geometric parameters (Å, º)

**Table:**

O1—C5	1.2322 (17)	N4—C2	1.3112 (18)
O2—C5	1.2560 (16)	N4—H4D	0.8600
O3—C6	1.2093 (17)	N4—H4E	0.8600
O4—C6	1.2913 (16)	N5—C3	1.3104 (18)
O4—H4	0.831 (10)	N5—H5A	0.8600
N1—C1	1.3054 (18)	N5—H5B	0.8600
N1—C2	1.3727 (17)	C1—C4	1.4812 (19)
N2—C3	1.3329 (17)	C4—H4A	0.9600
N2—C2	1.3400 (17)	C4—H4B	0.9600
N3—C1	1.3465 (17)	C4—H4C	0.9600
N3—C3	1.3643 (18)	C5—C6	1.5476 (19)
N3—H3	0.950 (19)		
			
C6—O4—H4	113.8 (16)	N2—C2—N1	124.86 (12)
C1—N1—C2	116.03 (12)	N5—C3—N2	120.67 (12)
C3—N2—C2	116.42 (11)	N5—C3—N3	118.55 (12)
C1—N3—C3	119.67 (12)	N2—C3—N3	120.76 (12)
C1—N3—H3	122.3 (11)	C1—C4—H4A	109.5
C3—N3—H3	117.8 (11)	C1—C4—H4B	109.5
C2—N4—H4D	120.0	H4A—C4—H4B	109.5
C2—N4—H4E	120.0	C1—C4—H4C	109.5
H4D—N4—H4E	120.0	H4A—C4—H4C	109.5
C3—N5—H5A	120.0	H4B—C4—H4C	109.5
C3—N5—H5B	120.0	O1—C5—O2	127.05 (13)
H5A—N5—H5B	120.0	O1—C5—C6	118.95 (12)
N1—C1—N3	122.20 (12)	O2—C5—C6	113.99 (12)
N1—C1—C4	119.85 (12)	O3—C6—O4	126.24 (13)
N3—C1—C4	117.95 (12)	O3—C6—C5	121.73 (12)
N4—C2—N2	119.38 (12)	O4—C6—C5	112.03 (11)
N4—C2—N1	115.75 (12)		
			
C2—N1—C1—N3	−0.4 (2)	C2—N2—C3—N5	−179.22 (13)
C2—N1—C1—C4	178.65 (13)	C2—N2—C3—N3	−1.0 (2)
C3—N3—C1—N1	1.8 (2)	C1—N3—C3—N5	177.20 (13)
C3—N3—C1—C4	−177.27 (13)	C1—N3—C3—N2	−1.1 (2)
C3—N2—C2—N4	−178.67 (14)	O1—C5—C6—O3	−166.01 (14)
C3—N2—C2—N1	2.5 (2)	O2—C5—C6—O3	13.0 (2)
C1—N1—C2—N4	179.32 (13)	O1—C5—C6—O4	13.65 (18)
C1—N1—C2—N2	−1.8 (2)	O2—C5—C6—O4	−167.33 (12)

### Hydrogen-bond geometry (Å, º)

**Table:**

*D*—H···*A*	*D*—H	H···*A*	*D*···*A*	*D*—H···*A*
N3—H3···O1^i^	0.95 (2)	1.77 (2)	2.7134 (17)	174 (2)
N3—H3···O4^i^	0.95 (2)	2.50 (2)	2.9841 (17)	111.7 (16)
O4—H4···O2	0.83 (2)	1.66 (2)	2.4921 (16)	175 (2)
N4—H4*D*···O3^ii^	0.86	2.17	2.9902 (19)	160
N4—H4*E*···N1^iii^	0.86	2.18	3.0399 (19)	174
N5—H5*A*···N2^iv^	0.86	2.14	3.0027 (19)	179
N5—H5*B*···O2^v^	0.86	2.28	2.8558 (17)	124
N5—H5*B*···O3^v^	0.86	2.59	3.2337 (19)	133
C4—H4*C*···O1^vi^	0.96	2.49	3.339 (2)	148

Symmetry codes: (i) *x*, *y*, *z*−1; (ii) *x*−1, *y*, *z*; (iii) −*x*, −*y*, −*z*+1; (iv) −*x*+1, −*y*+1, −*z*+1; (v) −*x*+2, −*y*+1, −*z*+1; (vi) *x*−1, *y*, *z*−1.
